# Supplementary material for: HIVprotI: an integrated web based platform for prediction and design of HIV proteins inhibitors
Source: J Cheminform. 2018 Mar 9;10:12. doi: 10.1186/s13321-018-0266-y (PMC5845081; doi:10.1186/s13321-018-0266-y)
Supplement: Supplementary file 2 — Additional file 2. Source code of HIVProtI web server. [file 13321_2018_266_MOESM2_ESM.zip › HIVprotI_Source-code/hivprotI/weka-3-6-6/doc/weka/classifiers/evaluation/TwoClassStats.html]

TwoClassStats


---


|  |  |  |
| --- | --- | --- |
| **Overview** \| **Package** \| **Class** \| **Tree** \| **Deprecated** \| **Index** \| **Help** \| **Weka's home** | |  |
| **PREV CLASS**   NEXT CLASS | **FRAMES**    **NO FRAMES**     **All Classes** |  |
| SUMMARY: NESTED \| FIELD \| CONSTR \| METHOD | DETAIL: FIELD \| CONSTR \| METHOD |  |


---


## weka.classifiers.evaluation Class TwoClassStats

```
java.lang.Object
  weka.classifiers.evaluation.TwoClassStats
```

**All Implemented Interfaces:**: RevisionHandler

---

``` public class TwoClassStats extends java.lang.Object implements RevisionHandler ```

Encapsulates performance functions for two-class problems.

**Version:**
:   $Revision: 1.9 $

**Author:**
:   Len Trigg (len@reeltwo.com)

---

**Constructor Summary**

`TwoClassStats(double tp,
double fp,
double tn,
double fn)`
  
          Creates the TwoClassStats with the given initial performance values.


| **Method Summary** | |
| --- | --- |
| `ConfusionMatrix` | `getConfusionMatrix()`             Generates a `ConfusionMatrix` representing the current two-class statistics, using class names "negative" and "positive". |
| `double` | `getFallout()`             Calculate the fallout. |
| `double` | `getFalseNegative()`             Gets the number of positive instances predicted as negative |
| `double` | `getFalsePositive()`             Gets the number of negative instances predicted as positive |
| `double` | `getFalsePositiveRate()`             Calculate the false positive rate. |
| `double` | `getFMeasure()`             Calculate the F-Measure. |
| `double` | `getPrecision()`             Calculate the precision. |
| `double` | `getRecall()`             Calculate the recall. |
| `java.lang.String` | `getRevision()`             Returns the revision string. |
| `double` | `getTrueNegative()`             Gets the number of negative instances predicted as negative |
| `double` | `getTruePositive()`             Gets the number of positive instances predicted as positive |
| `double` | `getTruePositiveRate()`             Calculate the true positive rate. |
| `void` | `setFalseNegative(double fn)`             Sets the number of positive instances predicted as negative |
| `void` | `setFalsePositive(double fp)`             Sets the number of negative instances predicted as positive |
| `void` | `setTrueNegative(double tn)`             Sets the number of negative instances predicted as negative |
| `void` | `setTruePositive(double tp)`             Sets the number of positive instances predicted as positive |
| `java.lang.String` | `toString()`             Returns a string containing the various performance measures for the current object |

**Methods inherited from class java.lang.Object**

`equals, getClass, hashCode, notify, notifyAll, wait, wait, wait`

**Constructor Detail**

### TwoClassStats

```
public TwoClassStats(double tp,
                     double fp,
                     double tn,
                     double fn)
```

:   Creates the TwoClassStats with the given initial performance values.

    **Parameters:**: `tp` - the number of correctly classified positives: `fp` - the number of incorrectly classified negatives: `tn` - the number of correctly classified negatives: `fn` - the number of incorrectly classified positives


**Method Detail**

### setTruePositive

```
public void setTruePositive(double tp)
```

:   Sets the number of positive instances predicted as positive

---


### setFalsePositive

```
public void setFalsePositive(double fp)
```

:   Sets the number of negative instances predicted as positive

---


### setTrueNegative

```
public void setTrueNegative(double tn)
```

:   Sets the number of negative instances predicted as negative

---


### setFalseNegative

```
public void setFalseNegative(double fn)
```

:   Sets the number of positive instances predicted as negative

---


### getTruePositive

```
public double getTruePositive()
```

:   Gets the number of positive instances predicted as positive

---


### getFalsePositive

```
public double getFalsePositive()
```

:   Gets the number of negative instances predicted as positive

---


### getTrueNegative

```
public double getTrueNegative()
```

:   Gets the number of negative instances predicted as negative

---


### getFalseNegative

```
public double getFalseNegative()
```

:   Gets the number of positive instances predicted as negative

---


### getTruePositiveRate

```
public double getTruePositiveRate()
```

:   Calculate the true positive rate.
    This is defined as

    ```
     correctly classified positives
     ------------------------------
           total positives
    ```

    :   **Returns:**: the true positive rate

---


### getFalsePositiveRate

```
public double getFalsePositiveRate()
```

:   Calculate the false positive rate.
    This is defined as

    ```
     incorrectly classified negatives
     --------------------------------
            total negatives
    ```

    :   **Returns:**: the false positive rate

---


### getPrecision

```
public double getPrecision()
```

:   Calculate the precision.
    This is defined as

    ```
     correctly classified positives
     ------------------------------
      total predicted as positive
    ```

    :   **Returns:**: the precision

---


### getRecall

```
public double getRecall()
```

:   Calculate the recall.
    This is defined as

    ```
     correctly classified positives
     ------------------------------
           total positives
    ```

    (Which is also the same as the truePositiveRate.)

    :   **Returns:**: the recall

---


### getFMeasure

```
public double getFMeasure()
```

:   Calculate the F-Measure.
    This is defined as

    ```
     2 * recall * precision
     ----------------------
       recall + precision
    ```

    :   **Returns:**: the F-Measure

---


### getFallout

```
public double getFallout()
```

:   Calculate the fallout.
    This is defined as

    ```
     incorrectly classified negatives
     --------------------------------
       total predicted as positive
    ```

    :   **Returns:**: the fallout

---


### getConfusionMatrix

```
public ConfusionMatrix getConfusionMatrix()
```

:   Generates a `ConfusionMatrix` representing the current
    two-class statistics, using class names "negative" and "positive".

    :   **Returns:**: a `ConfusionMatrix`.

---


### toString

```
public java.lang.String toString()
```

:   Returns a string containing the various performance measures
    for the current object

    :   **Overrides:**: `toString` in class `java.lang.Object`

---


### getRevision

```
public java.lang.String getRevision()
```

:   Returns the revision string.

    :   **Specified by:**: `getRevision` in interface `RevisionHandler`
    :   **Returns:**: the revision


---


|  |  |  |
| --- | --- | --- |
| **Overview** \| **Package** \| **Class** \| **Tree** \| **Deprecated** \| **Index** \| **Help** \| **Weka's home** | |  |
| **PREV CLASS**   NEXT CLASS | **FRAMES**    **NO FRAMES**     **All Classes** |  |
| SUMMARY: NESTED \| FIELD \| CONSTR \| METHOD | DETAIL: FIELD \| CONSTR \| METHOD |  |


---
